# Chemical quantification of *N*-acyl alanine methyl ester (NAME) production and impact on temporal gene expression patterns in *Roseovarius tolerans* EL-164

**DOI:** 10.1186/s12866-024-03624-7

**Published:** 2024-11-21

**Authors:** Janina Leinberger, Diana Koteska, Judith Boldt, Jörn Petersen, Sahana Shivaramu, Jürgen Tomasch, Stefan Schulz, Thorsten Brinkhoff

**Affiliations:** 1 Institute for Chemistry and Biology of the Marine Environment, University of Oldenburg, Oldenburg, Germany; 2 Institute of Organic Chemistry, Technische Universität Braunschweig, Braunschweig, Germany; 3 Leibniz Institute DSMZ - German Collection of Microorganisms and Cell Cultures, Braunschweig, Germany; 4 German Center for Infection Research (DZIF), Partner Site Braunschweig-Hannover, Braunschweig, Germany; 5 Laboratory of Anoxygenic Phototrophs, Institute of Microbiology of the Czech Acad Sci, Třeboň, 37981 Czechia

**Keywords:** Secondary metabolites, Signaling, Antibiosis, Roseobacter, Growth phases, Quantification, Transcriptomics, Differential gene expression, Time course

## Abstract

**Background:**

Previous studies have identified structurally diverse *N*-acyl amino acid methyl esters (NAMEs) in culture extracts of *Roseovarius tolerans* EL-164 (*Roseobacteraceae*). NAMEs are structural analogues of the common signaling compounds *N*-acyl homoserine lactones (AHLs), but do not participate in AHL-mediated signaling. NAMEs show minor antialgal and antimicrobial activity, but whether this activity serves as the primary ecological role remains unclear.

**Results:**

To enable dose-dependent bioactivity-testing, we have established a chromatographic method for quantification of NAMEs in bacterial culture extracts. The concentrations determined for the two major NAMEs produced by EL-164, C16:1-NAME and C17:1-NAME, ranged between 0.685 and 5.731 mg L^− 1^ (2.0-16.9 µM) and 5.3–86.4 µg L^− 1^ (15.0-244.3 nM), respectively. Co-quantification of the C14:1-AHL showed concentrations ranging between 17.5 and 58.7 mg L^− 1^ (56.6-189.7 µM). We observed distinct production patterns for NAMEs and AHLs, with a continuous NAME production during the entire incubation period. We conducted a spike-in experiment, using the determined metabolite concentrations. By comparing the transcriptomes of pre- and post-metabolite-spikes, we identified three clusters of differentially expressed genes with distinct temporal expression patterns. Expression levels of stress response genes differed between NAME- and AHL-spiked EL-164 cultures in the stationary phase.

**Conclusions:**

Our findings support previous studies suggesting an ecological role for C16:1-NAME as antibiotic, by proving that NAME concentrations in batch cultures were higher than the minimal inhibitory concentrations against *Maribacter* sp. 62 − 1 (*Flavobacteriia*) and *Skeletonema costatum* CCMP 1332 (*Coscinodiscophyceae*) reported in the literature. Our study further exemplified the broad application range of dose-dependent testing and highlighted the different biological activities of NAMEs and AHLs.

**Supplementary Information:**

The online version contains supplementary material available at 10.1186/s12866-024-03624-7.

## Introduction

Members of the Roseobacter group (synonyms: roseobacters, *Roseobacteraceae*) are among the most abundant bacteria (up to 20% in coastal habitats) in marine environments [1, 2]. Due to their substantial contributions to carbon and sulfur biogeochemical cycles, they are of vital importance to the global marine ecosystem [3, 4]. The broad distribution of roseobacters is partially attributed to their high metabolic flexibility [2], which includes the production of various bioactive secondary metabolites like antagonistic [5, 6] or signaling compounds [7, 8] but also a variety of so far uncharacterised molecules [9, 10]. *N*-Acyl homoserine lactones (AHLs) are signaling compounds most commonly found in Gram-negative bacteria [4, 7, 11]. In a density-dependent manner (*quorum sensing*, QS), AHLs regulate gene expression of a variety of genetically encoded traits including virulence factors, motility, biofilm formation and antibiotic production in representatives of the genera *Phaeobacter*, *Ruegeria*, *Dinoroseobacter* and other members of the Roseobacter group [12–14]. AHLs consist of a homoserine lactone ring linked via an amide bond to an acyl side chain [13]. The most common AHL derivatives in roseobacters carry monounsaturated side chains with 14, 16 or 18 carbon atoms [7]. The biosynthesis and mode of action of AHLs has been elucidated and described previously [15–17]. In brief, AHLs are synthesized from an acyl-acyl carrier protein (ACP) and S-adenosyl methionine (SAM) by a LuxI-type synthase and, depending on molecule size and polarity, can either diffuse freely across the cell membrane or are actively exported [18, 19]. Via uptake of own or foreign AHLs into the cell and subsequent binding to a corresponding LuxR-type transcriptional regulator, a transcription cascade is induced upon a threshold concentration [18].

In the past few years, structural analogues of AHLs, *N*-acyl amino acid methyl esters (NAMEs), have been found in several *Roseovarius* and *Loktanella* species of the Roseobacter group [7, 20]. In addition to the variations in side chain length and saturation found in AHLs, NAMEs are structurally even more diverse by the incorporation of different amino acids like valine, guanine and alanine, the latter being the predominant derivative in previously investigated strains [20]. Despite their striking structural resemblance to AHLs, NAMEs neither interfere with, nor inhibit AHL-based QS [21], but have been reported to exhibit minor algicidal and antibacterial properties against the centric diatom *Skeletonema costatum* [7] and diverse bacteria, respectively [7, 21]. Whether this bioactivity reflects the primary role of NAMEs in roseobacter ecology remains to be determined. The identification of the genetic basis for NAME synthesis has proven difficult and no candidate genes for the synthase have been found in previous studies [22]. The search for potential ecological roles and biosynthesis pathways of NAMEs may require comparisons with other, structurally similar bacterial metabolites like AHLs.

In this study, we conducted two main experiments. Firstly, we quantified the major NAMEs (here and in the following, the abbreviation refers to the *N*-acyl *alanine* methyl esters) produced by *Roseovarius tolerans* EL-164 at different growth stages, as basis for future, concentration-dependent investigations. In particular, the co-production of NAMEs and AHLs was investigated in this study. Secondly, we cultivated EL-164 in the presence of defined concentrations of external NAME, AHL or both. Transcriptomes of the metabolite-spike cultures were compared at different time points with regard to different distinct gene expression patterns.

## Materials and methods

### General experimental conditions and equipment

Chemicals (i.e., substrates used in chemical reactions) were purchased from Sigma Aldrich and used without further purification. Other solvents (used for purification or during chromatography) were purified by distillation and dried according to standard methods. Reactions with air- and moisture-sensitive compounds were carried out in vacuum-heated flasks under N_2_-atmosphere. Solutions were cooled to 0 °C in an ice-water bath. Thin layer chromatography was carried out on silica gel coated films (Polygram^®^ SIL G/UV254, layer thickness 0.2 mm; Macherey-Nagel, Düren, Germany). In addition to UV-detection (254 nm), common staining reagents such as molybdophosphoric acid or potassium permanganate were used. Flash column chromatography was carried out on silica gel 60 Å (grain size 35–70 μm; Fisher Scientific, Waltham, MA, USA). NMR spectra were recorded at room temperature with a Bruker AVANCE III 400 spectrometer (400 MHz for ^1^H, 100 MHz for ^13^C). Chemical shifts are given in ppm relative to the internal standard tetramethylsilane. The following abbreviations are used to indicate the multiplicities of the respective signals: s (singlet), d (doublet), t (triplet), quin (quintet), m (multiplet), and hept (heptett). The coupling constants *J* are given in Hertz (Hz). The ^13^C NMR spectra are ^1^H broadband decoupled spectra.

GC/MS-analyses of IS-spiked extracts from EL-164 cultures (quantification experiment, Supplement F1, F2) were carried out on a GC 7890 A system connected to a 5975 C mass-selective detector and equipped with a 7683B injector (all components from Agilent Technologies, Santa Clara, CA, USA). The separation was performed on a fused silica capillary column HP-5MS (30 m × 0.25 mm I.D. × 0.25 μm film, Agilent Technologies). Helium was used as carrier gas (flow rate of 1.2 mL/min). Electron impact ionization was performed at 70 eV. Analyses of culture extracts were carried out in splitless mode at the following temperature program: start temperature 50 °C (5 min. isothermal), heating rate 5 °C/min, final temperature 320 °C (10 min isothermal). The GC conditions were as follows: inlet pressure 67.5 kPa, He 24.2 mL/min, injection volume 1 µL, injector 250 °C, transfer line 280 °C. An automatic sampler used for injections was set to the following conditions: syringe size 10 µL, injection volume 1 µL, sample washes 2x, sample wash volume 3 µL, sample pumps 5x. The quantification was proceeded with the software *Enhanced Data Analysis* (Agilent).

### Synthesis of chemical components and chromatographic analysis of selected *R. tolerans* EL-164 metabolites

#### Synthesis of deuterated l-alanine methyl ester hydrochloride (2) 

**Fig. 1:**
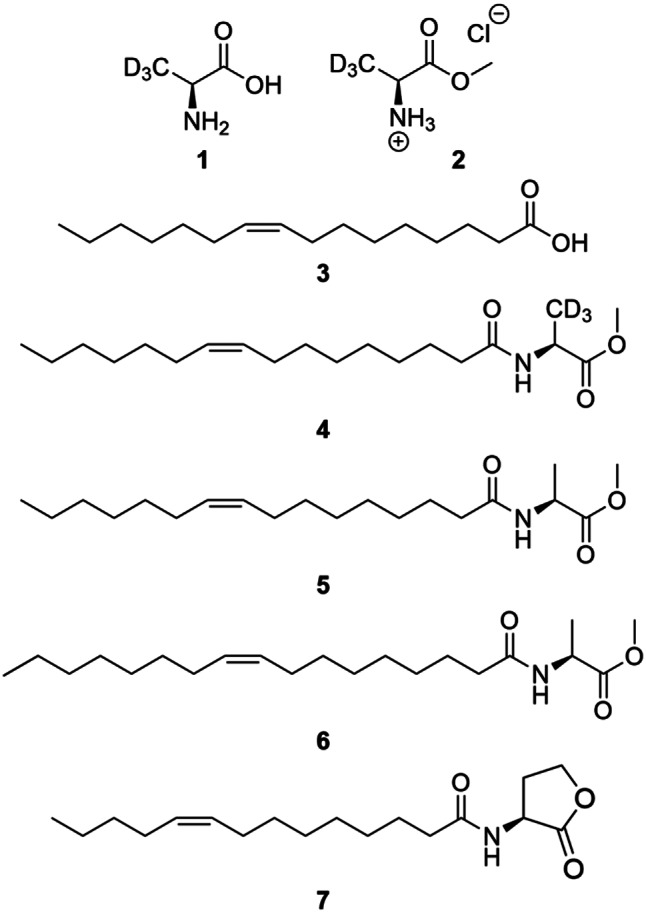
Chemical structures of EL-164 metabolites and reagents used for synthesis of the internal standard. Components **1**–**3** were used for synthesis of the internal standard **4**. Compounds **5**–**7** are natural metabolites of *R. tolerans* EL-164 and were measured in culture extracts via GC/MS. **1** l-alanine-d_3_, **2** lalanine methyl ester hydrochloride, **3** palmitoleic acid, **4** internal standard C16:1-NAME-d3, **5** natural C16:1-NAME, **6** natural C17:1-NAME and **7** C14:1-AHL

A solution of l-alanine-*d*_3_ (**1**, 500 mg, 5.43 mmol, 1.00 eq., Sigma Aldrich, St. Louis, MO, USA) in methanol (6.0 mL) was cooled to 0 °C and thionyl chloride (904 mg, 0.55 mL, 7.60 mmol, 1.40 eq.) was added. The mixture was stirred for 6 h at reflux and then for 16 h at room temperature. The crude product was filtered over celite to remove excess reagent 1. The solvent was removed under reduced pressure and the product was dried at high vacuum. Compound **2** (764 mg, 2.35 mmol, quant.) was obtained as a pale yellow crystalline solid. The synthesis was carried out as described previously [23]. Chemical structures are shown in Fig. 1.

^**1**^**H-NMR** (500 MHz, DMSO): *δ* = 8.74 (s, 3 H), 4.03 (s, 1 H), 3.74 (s, 3 H).

^**13**^**C-NMR** (125 MHz, DMSO): *δ* = 170.3, 52.7, 47.5, 14.9 (hept., *J* = 19.9 Hz).

### Synthesis of the internal standard Z9-C16:1-NAME-d3 (4) 

A solution of palmitoleic acid (**3**, 200 mg, 0.22 mL, 0.79 mmol, 1.00 eq.) in dry dichloromethane (DCM, 10 mL) and a drop of *N*,*N*-dimethylformamide (DMF) was cooled to 0 °C. Oxalyl chloride (151 mg, 0.10 mL, 1.19 mmol, 1.50 eq.) was slowly added and the mixture was stirred until gas formation ceased. The solvent was removed under reduced pressure to obtain the acid chloride. After slow addition of triethylamine (320 mg, 0.44 mL, 3.16 mmol, 4.00 eq.) to a solution of deuterated l-alanine methyl ester hydrochloride (**2**, 170 mg, 1.19 mmol, 1.50 eq.) in dry DCM (10 mL), the mixture was stirred for 5 min and the acid chloride, dissolved in DCM (10 mL), was added to the mixture. After stirring for 3 h at room temperature, ethyl acetate (50 mL) was added. The organic phase was washed twice, with saturated NaHCO_3_-sol. and with H_2_O, and dried over MgSO_4_. The solvent was removed under reduced pressure and the crude product was purified by column chromatography on silica gel (pentane/ethyl acetate 4:1) to obtain the deuterated compound **4** (272 mg, 0.79 mmol, quant.) as a clear oil. The procedure was carried out as described [21].

*R*_f_: 0.24 (pentane/ethyl acetate 4:1).

*R*_I_: 2473.

^1^H-NMR (500 MHz, CDCl_3_): *δ* = 6.09 (d, *J* = 6.4 Hz, 1 H), 5.38–5.30 (m, 2 H), 4.59 (d, *J* = 7.4 Hz, 1 H), 3.75 (s, 3 H), 2.21 (t, *J* = 7.6 Hz, 2 H), 2.04–1.96 (m, 4 H), 1.63 (quin, *J* = 7.3 Hz, 2 H), 1.37–1.22 (m, 16 H), 0.88 (t, *J* = 6.9 Hz, 3 H).

^13^C-NMR (125 MHz, CDCl_3_): *δ* = 173.7, 172.6, 129.9, 129.7, 52.4, 47.6, 36.5, 31.7, 29.7, 29.6, 29.2, 29.1 (2x), 28.9, 27.2, 27.1, 25.5, 22.6, 17.7 (hept., *J* = 19.8 Hz), 14.1.

MS (EI, 70 eV): *m*/*z* (%) = 342 (5) [M]^+^, 283 (3), 161 (8), 148 (14), 129 (3), 108 (5), 107 (100), 106 (3), 61 (3), 47 (60).

### Origin and cultivation of *Roseovarius tolerans* EL-164

*R. tolerans* EL-164 had been previously isolated from the hypersaline Ekho Lake in Antarctica [24] and is available under the accession number DSM 108135 in the culture collection of the German Collection of Microorganisms and Cell Cultures GmbH (DSMZ, Braunschweig, Germany). Aliquots from cryo stocks of EL164 prepared in our lab were first transferred on agar plates and incubated overnight at room temperature prior to inoculation of liquid cultures from a single colony. Agar plates were prepared with 1.7% (/v) agar. If not stated otherwise, the strain was routinely cultivated in marine broth (MB) medium with modifications described previously [12]. Incubations were carried out in the dark, at 20 °C and 100 rpm. Growth was monitored by measuring the optical density at 600 nm (OD_600nm_). Two main experiments were conducted in this study: Firstly, NAMEs and AHLs were extracted from EL-164 cultures to chromatographically determine their concentrations in the bacterial cultures. Secondly, a spike-in experiment with synthetic C16:1-NAME and C14:1-AHL was conducted, using the metabolite concentrations determined in the quantification experiment. In this second experiment, RNA was extracted before and after spike-in of metabolites for subsequent comparative transcriptomics. The workflows of both experiments are briefly described in the following. The full description of the experimental setups can be found in Supplement F1.

### Quantification experiment: extraction of NAMEs and AHLs from EL-164 cultures

Several pre-experiments were conducted to identify a suitable extraction method for NAMEs. The tested parameters were (i) if and when an adsorbent should be added to the bacterial culture and (ii) at which time point the internal standard (IS) **4** should be added (Supplement F1). Based on the obtained results, a liquid-liquid extraction procedure was chosen for the quantification experiment described in this study. The IS was added to each sample directly after removal from EL-164 cultures. All tested parameters and corresponding results are described in detail in Supplement F1.

For metabolite extraction, the bacterial culture was grown under routine conditions. A sample was aseptically removed from the culture and the IS was added before the sample was centrifuged (7,500 rpm, 4 °C, 30 min). Subsequently, the supernatant (SN) was sterile-filtered (ø 2 μm, PES, Fisher Scientific, Waltham, MA, USA) and extracted with DCM by mixing in a separatory funnel (DCM: SN = 1:3). The aqueous and the organic phase were collected separately. The aqueous phase was re-extracted twice with DCM. After the third extraction step, the aqueous phase was discarded and the collected organic extract was dried with anhydrous MgSO_4_ and filtered through a paper filter (Rotilabo, type 600P). The organic extract was concentrated in a rotary evaporator (Multivapor P-6, Büchi, Flawil, Switzerland) and stored at -20 °C until chemical analysis.

### Isotope dilution analysis

Extracts obtained after the addition of the IS **4** were analyzed by GC/MS and the peak areas of diagnostic ions were quantified using the respective ion traces. The characteristic base peaks at *m/z* 104 (Z9-C16:1-NAME, **5**) and 107 (Z9-C16:1-NAME-d_3_, **4**) served as these quantifier ions. Their peak area ratio was used for the calculation of the concentration of NAME **5** from the predefined concentration of IS **4** (210 µg L^− 1^). The quantification of the NAME was adapted analogous to the previously described procedure for AI-2 quantification [35]. Due to the shorter C-D-bond length compared to the C-H bond length (Supplement F2, Figure S1), the IS eluted slightly earlier than the unlabeled compound. Therefore, peak areas were combined for quantification. The IS was also used for quantification of C17:1-NAME (**6**), leading to less accurate results for this compound. Finally, C14:1-AHL (**7**) was quantified by comparing their peak area with that of the IS.

### Spike-in experiment: RNA extraction, library preparation, sequencing and bioinformatic analysis

In the spike-in experiment (sampling time points t_S1−S3_) EL-164 cultures were spiked with C16:1-NAME (**6**) (at 24 h), C14:1-AHL (**7**) (at 49 h) or both metabolites (at 24 h and 49 h, respectively). Sampling for RNA sequencing was conducted at 26 h (t_S1_), 49 h (t_S2_) and 53 h (t_S3_). For RNA sampling, 2 ml EL-164 culture were removed aseptically, snap frozen in liquid nitrogen and subsequently stored at -80 °C until RNA extraction. The latter was performed as described earlier [25], omitting the DNA extraction steps.

### Sequencing and data quality control

Illumina libraries were generated as described previously [26], including rRNA removal with the RiboZero Kit (Illumina, USA) and sequenced on a NovaSeq 6000 (Illumina, USA) in paired-end mode with 100 cycles in total. Raw reads were processed, and differential gene expression was assessed as described before [27]. Briefly, reads were filtered with fastp (version 0.20.1; [28]) and mapped to the *R. tolerans* EL-164 genome (Genbank accession GCF_028885395.1) using BWA-MEM (version 0.7.12; [29]) and SAMtools (version 0.1.19; [30]). HTSeq-count (version 0.13.5; [31]) was used to assess the number of reads per gene. Normalization and identification of significantly differentially expressed genes (false discovery rate, FDR < 0.05) was performed with DESeq2 (version 1.32.0; [32]) using the likelihood ratio test with the growth time and condition as the full model, and as the reduced model only the growth time. For assessing the quality of our data, we used a principal component analysis in R.

### Identification of relevant gene expression patterns in EL-164 spike-in cultures

Z-score normalization was used over the replicates and conditions of each significantly differentially expressed gene to compare the transcription patterns of genes with different absolute transcription levels. Subsequently, the genes were clustered by their z-scores using a hierarchical clustering in python (scipy.cluster.hierarchy.linkage with average distance and citiblock metric; scipy.cluster.hierarchy.fcluster with k = 3 and maxclust criterion).

### Functional analysis of metabolite-spiked EL-164 cultures

Potential metabolic functions were assigned to significantly differentially expressed genes (DE genes) using COG categories (Clusters of Orthologous Groups of proteins) and GO processes (Gene Ontology) assigned by eggNOG mapper [33, 34] together with the GO annotation provided by NCBI.

## Results

### Quantification of NAMEs via isotope dilution analysis

**Fig. 2:**
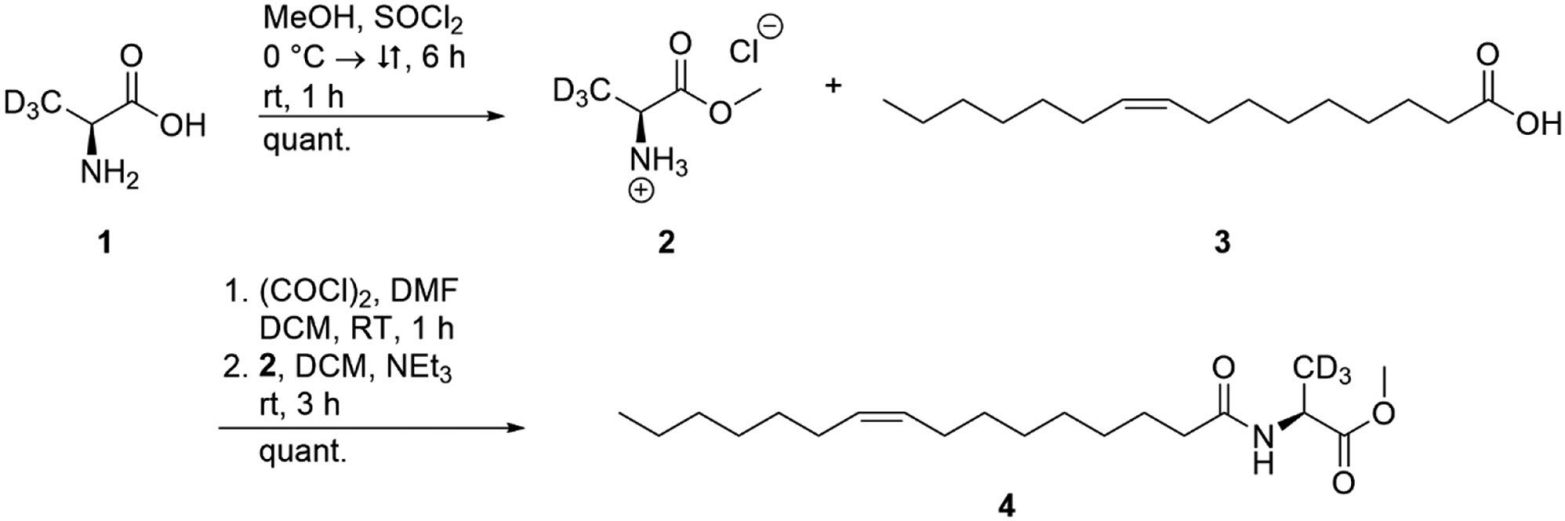
Synthesis of the internal standard for quantification of NAMEs in bacterial culture extracts. Synthesis of the internal standard C16:1-NAME-d_3_ (**4**) from l-alanine–d_3_ (**1**), via deuterated methylester hydrochloride (**2)** and amide coupling with palmitoleic acid (**3**) ]

In the first experiment (quantification experiment, sampling time points t_Q0−5_), NAMEs **5** and **6** were quantified in *R. tolerans* EL-164 culture extracts by GC/MS using isotope dilution analysis with an isotopically labeled IS. Here, a labeled version of the major NAME released by EL-164, (*Z*)-*N*-(hexadec-9-enoyl)alanine methyl ester (Z9-C16:1-NAME, **5**) [13], served as IS. This standard Z 9-C16:1-NAME-d_3_ (**4**) had to be synthesized. Because of its ready availability, alanine-*d*_3_ was selected as carrier of the label. The synthesis of the IS was carried out as follows: l-alanine-d_3_ (**1**) was converted into the deuterated methyl ester hydrochloride **2** with methanol and thionyl chloride (Fig. 2). Subsequent amide coupling with palmitoleic acid (**3**) in the presence of oxalyl chloride resulted in the deuterated NAME **4** with a quantitative yield.

**Fig. 3:**
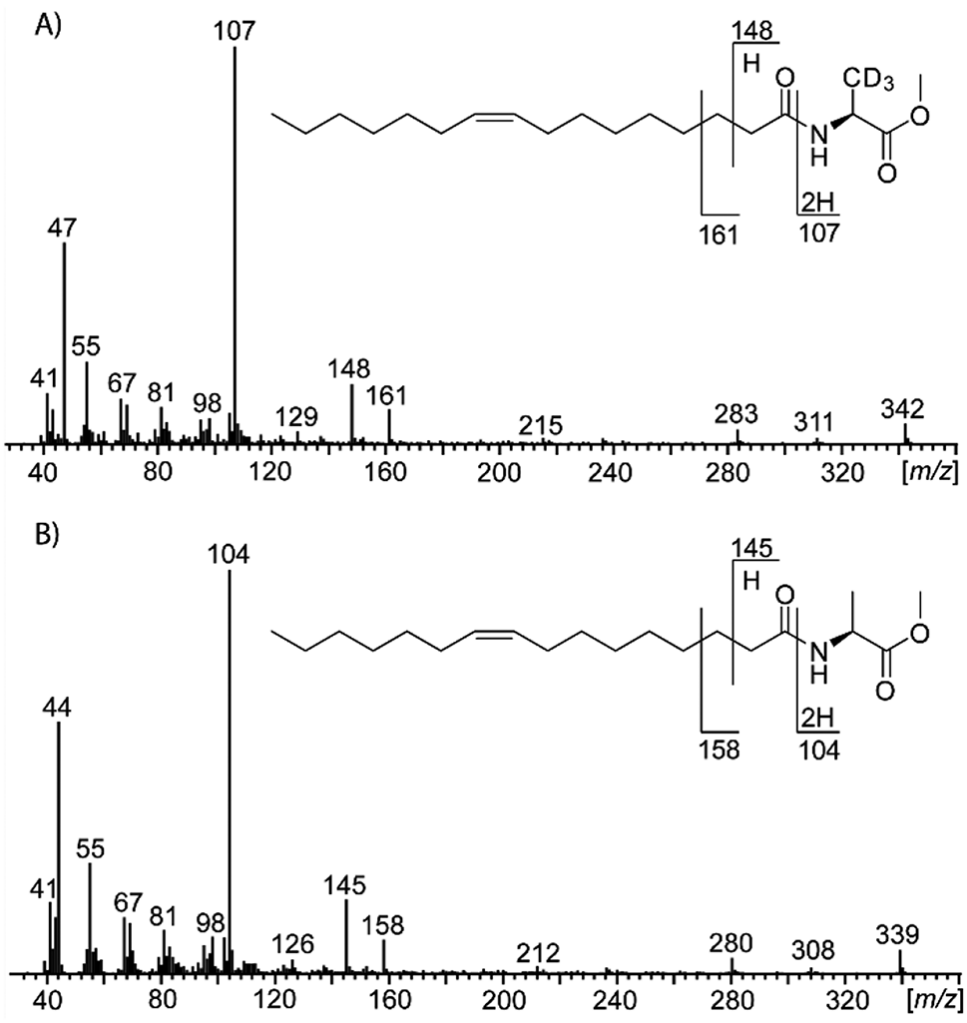
Mass spectra of C16:1-NAME and deuterated standard IS in EL-164 culture extracts. Mass spectra and fragmentations of **A**) the deuterated internal standard **4** and **B**) the natural metabolite **5**. The ratio of the base peaks at m/z 107 (**A**) and 104 (**B**) were used for quantification of **5**.

The mass spectra of the naturally occurring Z9-C16:1-NAME (**5**) and the labeled analogue **4** revealed characteristic base peaks at *m/z* 104 and 107, respectively (Fig. 3). These served as quantifier ions in the isotope dilution analysis. The quantification of the NAME was adapted analogously to the previously described procedure for AHL quantification [35].

### Quantification of NAMEs and AHLs in different growth phases of *R. tolerans* EL-164 reveals distinct production patterns

**Fig. 4:**
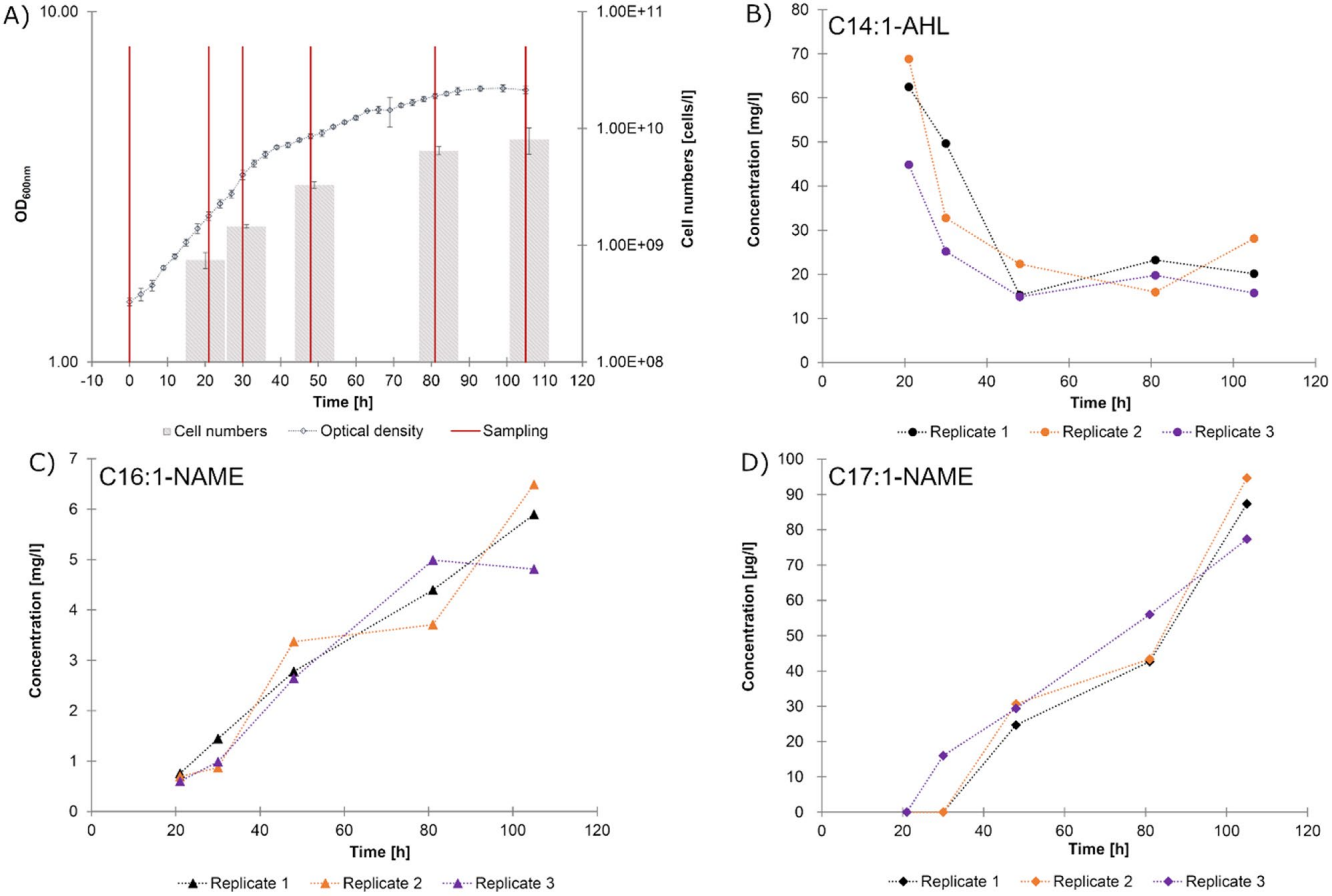
Growth of EL-164 and metabolite concentrations determined during the quantification experiment. (**A**) Growth of EL-164 over time displayed via OD600nm measurements and cell numbers, as well as sampling time points. Samples were removed from cultures at several time points (indicated with vertical lines are t_Q05_, 0–105 h) for quantification of NAMEs and AHLs and determination of cell counts (indicated by grey bars). OD600nm values and cell numbers are means from biological triplicates. Concentrations of (**B**) C14:1-AHL (**7**), (**C**) C16:1-NAME (**5**). and (**D**) C17:1-NAME (**6**) determined at time points t_Q15_ (21–105 h) in bacterial culture extracts

An initial growth experiment was conducted for quantification of NAMEs and AHLs in EL-164 cultures at different time points. Under standard growth conditions, EL-164 reached the stationary phase after t_Q4_ (81 h). The observed increase in cell number was significant between t_Q0_-t_Q4_ (0–81 h), but not between t_Q4_-t_Q5_ (81–106 h, p_tQ4,tQ5_=0.315). This supports our assumption (deduced from the decreasing growth rates) that growth of EL-164 decelerated between t_Q3_-t_Q4_ (48–81 h) and the strain entered the stationary phase. Cultures were further incubated until t_Q5_ (105 h) without observation of a beginning death phase (Fig. 4A).

The IS C16:1-NAME-d_3_ (**4**) was not detected in t_Q0_ samples. Therefore, quantification was not possible at t_Q0_ (0 h), but presence/absence of metabolites was still noted. C14:1-AHL (7), the main AHL of EL-164, was found in gas chromatograms of all culture extracts, i.e. all replicates from all time points (Supplement F2, Table S1). AHL concentrations ranged between 17.5 and 58.7 mg L^− 1^ (56.6-189.7 µM), were high in the mid-exponential growth phase and decreased thereafter (Fig. 4B). Conversely, C16:1-NAME concentrations were an order to magnitude lower (0.685–5.731 mg L^− 1^, 2.0-16.9 µM) and showed a steady increase between t_Q1_-t_Q5_ (21–105 h, Fig. 4C). While C17:1-NAME concentrations showed a similar trend as C16:1-NAME concentrations (continuous increase between t_Q2_-t_Q5_, Fig. 4D), the determined concentrations (5.3–86.4 µg L^− 1^, 15.0-244.3 nM) of C17:1-NAME were even lower than those of C16:1-NAME (Supplement F2, Table S1). In addition, C17:1-NAME was not found in samples from t_Q0_ (0 h) and t_Q1_ (21 h) and only in one of three replicates at t_Q2_ (30 h).

### Transcriptomic analysis of expression patterns in C16:1-NAME- and C14:1-AHL-spike-in cultures of *R. tolerans* EL-164

#### Temporal patterns of gene expression in metabolite-spiked EL-164 cultures

**Fig. 5:**
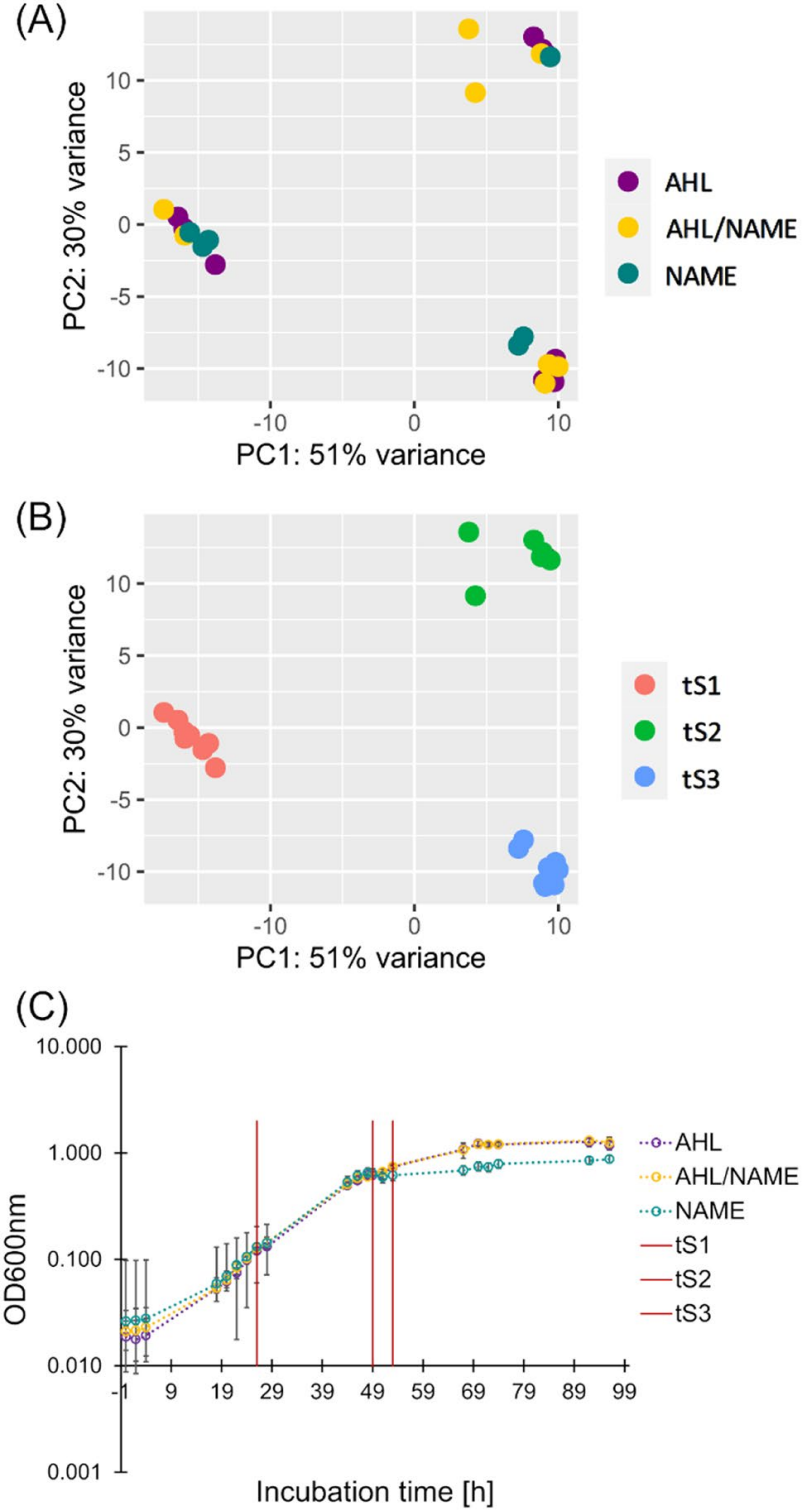
EL-164 transcriptome analysis and growth curve of EL-164 during the spike-in experiment. Gene expression profiles were analyzed via principal component analysis and colored according to (**A**) metabolite spike and (**B**) growth time. (**C**) Growth curve of EL-164 cultures spiked with C16:1-NAME (24 h), C14:1-AHL (49 h) or both metabolites. Sampling time points for RNA sequencing are indicated with red vertical lines

In addition to the quantification experiment, we conducted an experiment with spiked-in C16:1-NAME, C14:1-AHL or both metabolites. The time points for spiking and the concentrations were chosen based on the findings from the quantification experiment, when the concentrations of the respective metabolite were low in the batch culture (detailed description in Supplement F1). A principal component analysis (PCA) over the transcriptomes at the different time points and conditions showed clear separation along the growth times, but not along the conditions (Fig. 5A, B). Consequently, gene expression patterns over time were further investigated. The EL-164 growth curve including indication of sampling points is provided in Fig. 5C.

**Fig. 6:**
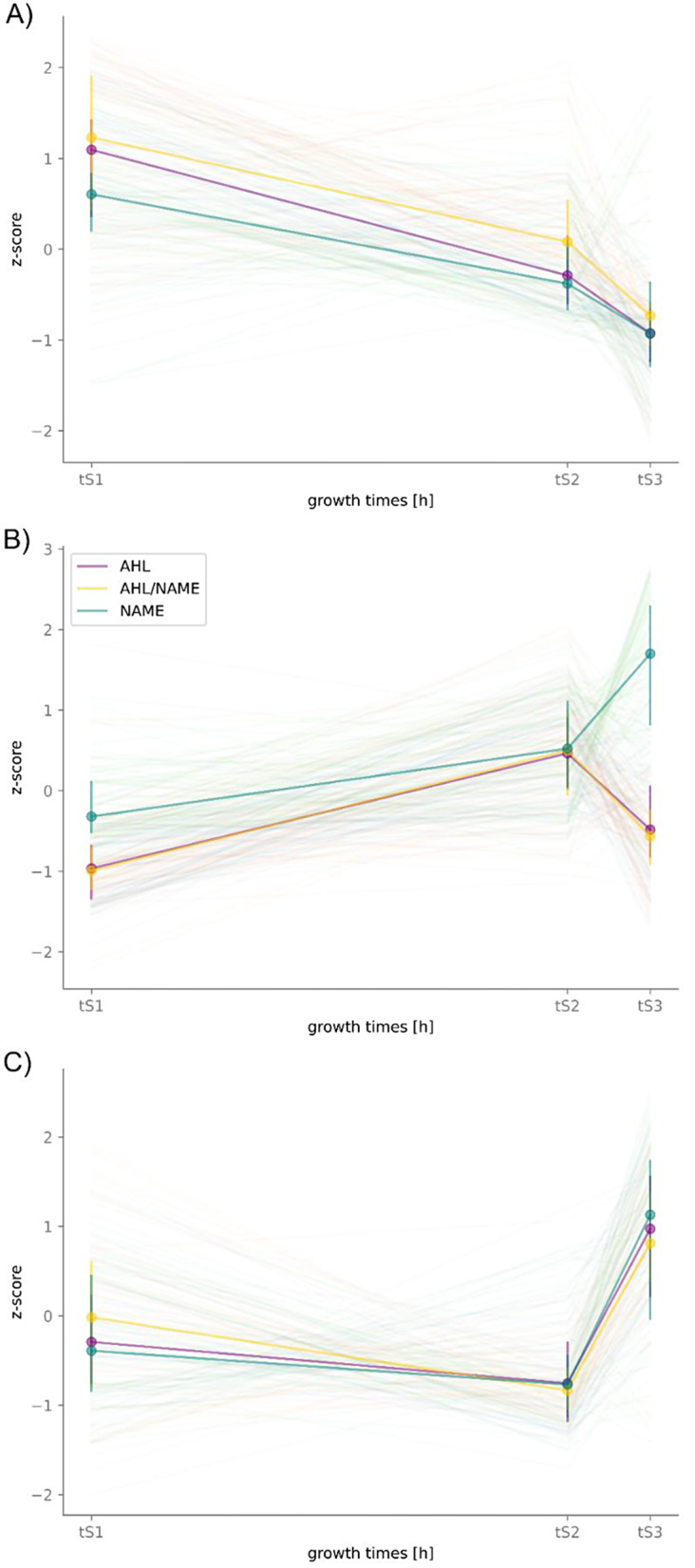
Temporal clusters of significantly differentially expressed genes determined in metabolite-spiked EL-164 cultures. Three different temporal patterns of significantly differentially expressed genes were observed (across the conditions AHL-spike, NAME-spike or AHL/NAME-spike). **A**) cluster 1, **B**) cluster 2 and **C**) cluster 3. Z-scores represent deviation from mean transcription. Thin lines represent individual genes and thick lines median values and interquartile ranges over all genes in a cluster. Color code: purple - C14:1-AHL-spiked cultures, green - C16:1-NAME-spiked cultures and yellow AHL- and NAME-spiked cultures

Using DESeq2 and the likelihood ratio test provided by the program, we identified 187 genes that were significantly differentially expressed (DE) across the conditions when simultaneously controlling for the effect of the growth time (Supplement F3). It is important to note, that we observed a high variability between biological replicates that was at times larger than the variability across conditions. For instance, we observed a larger variability between the NAME-spiked samples at t_S1_ than towards the non-spiked sample (AHL) (Fig. 6). Nevertheless, clear differences in temporal gene expression were visible between the conditions. To better evaluate the temporal expression of the 187 DE genes, we calculated their transcription patterns (z-scores) over all time points and conditions and grouped them via hierarchical clustering (Fig. 6). Cluster 1 comprised 62 genes (Fig. 6A), cluster 2 was the largest with 76 genes (Fig. 6B) and cluster 3 the smallest with 49 genes (Fig. 6C). Individual plots for temporal expression patterns were created for all DE genes and can be found in Supplement F3.

Within clusters 1 and 3 the median temporal patterns of the different treatments (AHL, AHL/NAME, NAME) were similar. Many genes in cluster 1 were maximally expressed at t_S1_ (26 h) and showed a decrease in expression at t_S2_ (49 h), with a more pronounced downward trend at t_S3_ (53 h, Fig. 6A). Conversely, the majority of genes in cluster 3 showed a strong increase in expression at t_S3_ compared to t_S1_ and t_S2_. Genes in cluster 2 exhibited distinct temporal transcription patterns. Most genes in this cluster showed a distinctly higher expression at t_S3_ under the NAMEs treatment than under the conditions with the AHL spikes (AHL and AHL/NAME). The reason for this strong variation between the conditions might be different for each gene in cluster 2 and could have been either an upregulation of a gene at t_S3_ by the NAME spiked at t_S1_, or a downregulation of a gene by the spiked AHL at t_S2_. Independent of the individual reason behind the temporal patterns, genes in cluster 2 were markedly differently affected by C16:1-NAME and C14:1-AHL.

### Functional analysis of the 187 differentially expressed genes

We assigned GO process terms (referred to as “GO terms” or “GO processes” in the following) and Cluster of Orthologous Groups (COG) categories to the 187 DE genes using eggNOG mapper [33, 34]. In total, 28/64, 24/76 and 9/49 GO terms were assigned to clusters 1, 2 and 3, respectively. COG categories were assigned to 57/64, 56/76 and 41/49 genes in clusters 1, 2 and 3, respectively (Supplement F3).

Cluster 1 was the best characterized by GO terms and COG categories and comprises only one hypothetical protein. Hence, genes in cluster 1 were well represented and described in reference databases. The most strongly represented COG categories corresponded to information storage and processing (17.7%) and metabolism (48.4%) with the major categories J (“Translation, ribosomal structure and biogenesis”) and EH (“Amino acid transport and metabolism”, “Coenzyme transport and metabolism”), respectively (Supplement F3). This coincided with the assigned GO terms that matched with translation, gene expression and biosynthesis processes. Genes in cluster 1 mostly corresponded to primary metabolism and encode components of ribosomal subunits (PVU19_RS06060, PVU19_RS07965, PVU19_RS08950, PVU19_RS18125), ubiquinone biosynthesis (PVU19_RS01000, PVU19_RS01250), diverse transporters (e.g., PVU19_RS02070, PVU19_RS02405, PVU19_RS02835, PVU19_RS03345, PVU19_RS05975, PVU19_RS06130) and the general regulator sigma factor 70 (PVU19_RS11495) (Supplement F3). As expected, these primary metabolic genes were upregulated in the exponential phase compared to the other growth phases, when cells proliferate rapidly (Fig. 6A).

Cluster 2 exhibited a lower amount of characterized GO terms and COG categories than cluster 1 and comprised 12 hypothetical genes (15.8%) (Supplement F3). The most abundant COG category was category S (“Function unknown”), indicating that a large number (22.4%) of the genes in cluster 2 was poorly characterized, despite their association to a COG category. This reflects an insufficient functional annotation and representation in available reference databases. Apart from the most abundant category of poorly characterized genes, the two next most abundant COG categories were associated with cellular processes and signaling (21.1%) and information storage and processing (15.8%). The most prominent categories were TUO (“Signal transduction mechanisms”, “Intracellular trafficking, secretion, and vesicular transport”, “Posttranslational modification, protein turnover, chaperones”) and L (“Replication, recombination and repair”), respectively (Supplement F3). GO terms throughout cluster 2 mostly matched with processes from cellular component assembly, protein metabolism, secretion and transport. Interestingly, cluster 2 comprised an array of genes involved in response to environmental stimuli: stress response protease HslVU (PVU19_RS00735, PVU19_RS00740), superoxide dismutase SodB (PVU19_RS08760), chaperone ClpA (PVU19_RS17865), heat-inducible repressor HrcA (PVU19_RS00855) and regulators LysR (PVU19_RS09385), YdiU (also known as SelO, PVU19_RS10705) and RbfA (PVU19_RS19205), potentially involved in stress response. Furthermore, genes involved in iron-sulfur cluster biosynthesis (PVU19_RS07615, PVU19_RS08920, PVU19_RS08925), taurine catabolism (PVU19_RS04020) and a type II secretion system (PVU19_RS00780, PVU19_RS02080, PVU19_RS05935, PVU19_RS05940) were found in cluster 2 (Supplement F3).

Cluster 3 had the least GO terms assigned. COG categories were mostly associated with metabolism (44.9%) and predominantly represented by category E (“Amino acid transport and metabolism”) (Supplement F3). COG categories assigned to cluster 3 genes showed a similar distribution compared to the distribution over the complete genome of EL-164 (Supplement F3). However, among metabolism-associated COG categories, category E were relatively prominent in cluster 3 as compared to the complete genome (Supplement F3 and Supplement F2, Figure S4), while category Q (“Secondary metabolites biosynthesis, transport and catabolism”) was not assigned to any gene within cluster 3. In coincidence with the assigned COG categories, GO processes were almost exclusively associated with protein metabolism and biosynthesis. In addition, several stress response genes were identified within cluster 3 encoding for the oxidative damage protectant rubrerythrin (PVU19_RS02855) and the regulators Crp/Fnr (PVU19_RS06840), MerR (PVU19_RS17190) and LysR (PVU19_RS18985).

## Discussion

The ecological role of most secondary metabolites is hitherto unknown. To better understand their purpose and infer their ecological roles, it is crucial to identify biologically relevant concentrations and potential growth phase-dependent production patterns. In this study, we focused on the known signaling molecule C14:1-AHL and its structural analogue, the potentially antagonistic compound C16:1-NAME in *R. tolerans* EL-164. To further differentiate the ecological roles of these structurally similar compounds, we compared their production patterns over different growth phases and studied the impact of both metabolites on temporal gene expression.

### Secondary metabolites C16:1-, C17:1-NAMEs and C14:1-AHL exhibit distinct production patterns

*R. tolerans* EL-164 exhibited peak AHL production during exponential growth, followed by subsequently decreasing concentrations towards the stationary phase. This pattern is typical for signaling compounds and was observed in other bacteria, including other *Roseobacteraceae* [36–38]. Compared to AHL concentrations determined in samples from *Pseudomonas* spp. (124 nM [37], max. 1.5 mM, *Gammaproteobacteria* [39]), *Serratia liquefaciens* (< 0.1–10 µM, *Gammaproteobacteria* [40]), *Burkholderia cepacia* (max. 1.7 µM, *Betaproteobacteria* [41]) and *Shewanella baltica* (10–80 µM, *Gammaproteobacteria* [38]) we consider AHL-production by EL-164 (*Alphaproteobacteria*) as relatively high (56.6-189.7 µM). Our findings corroborate previous studies that have not quantified AHLs in roseobacters but have demonstrated an effect of AHLs on biofilm formation, motility and gene expression at concentrations between 12 nM − 5 µM [14, 42–45].

In contrast to AHLs, NAMEs were produced by EL-164 throughout the incubation time of 105 h, including later growth stages (concentration range 2.0-16.9 µM). Based on the markedly differing temporal production patterns and concentrations (Fig. 4B-C), we assume that the metabolites serve distinct purposes. As opposed to signaling compounds, many antibiotics are produced throughout the entire growth phase, often reported to accumulate or increase in production in the late exponential to stationary growth phases [46]. Examples include the roseobacterial compound tropodithietic acid (TDA) [11] and bacteriocins [47]. The results also coincide with previous studies reporting weak antialgal and antibacterial activity of nanomolar C16:1-NAME concentrations [7, 20, 21]. However, the observed antibacterial activity range was very narrow: Growth inhibition was reported exclusively against *Maribacter* sp. 62 − 1 (*Flavobacteriia*) and the diatom *Skeletonema costatum* CCMP 1332 (*Coscinodiscophyceae*) [7, 22]. Both organisms are potential interaction partners in the natural habitat of *R. tolerans* EL-164. *Maribacter* spp. have been found in arctic marine environments [48] and several members of the class *Flavobacteriia* were co-isolated with EL-164 from Ekho Lake [49]. Due to its very limited target range, it remained questionable whether the antibiotic activity can be considered the primary function of C16:1-NAME. In the present study, we measured C16:1-NAME concentrations in EL-164 batch cultures that were one order of magnitude higher than the minimal inhibitory concentrations (MIC) defined earlier [7, 22]. Consequently, we assume that the antibiotic potential of C16:1-NAME might have been underestimated and re-testing at micromolar concentrations could also expand the known target range. Hence, our quantification method enables the validation of a potential antagonistic role of NAMEs via identification of biologically relevant concentrations and observation of production characteristics in a defined system. Such dose-dependent testing is of great importance, considering that known signaling compounds like AHLs can exhibit antibiotic activities at sufficiently high concentrations or, vice versa, antibiotics such as TDA can have signaling function at sub-inhibitory concentrations [12].

Our findings indicate a high potential for the discovery of new NAME-mediated biological and ecological roles, as we have observed distinct differences in C16:1- and C17:1-NAME production patterns and concentrations. Considering the structural diversity of N-acyl amino acid methyl esters produced by EL-164, including variations in chain length, amino acids, side chains and saturation, a comparison of the different derivatives regarding production characteristics and function is necessary. Previous studies have reported antimicrobial properties of NAME derivatives [7, 21], while other, structurally similar *N*-acylated amino acids exhibited a range of activities including surfactant [50], hemolytic [51] and potential transcriptional influence [52]. To further characterize potential roles of NAMEs and their derivatives, it would be interesting to determine intra- and extracellular NAME concentrations. Until now, all experiments were conducted with whole cell extracts. However, both fractions should be tested separately to determine how NAMEs interact with the extracellular environment. Ideally, such experiments would also consider the solubility of NAME in aqueous medium to identify potential distribution via diffusion (long- vs. short-distance effects). All experiments in this study were carried out in complex medium. However, nutrient availability has a strong impact on metabolite production characteristics [53, 54]. For instance, the maximum AHL concentration determined for *B. cepacia* was 40 times higher in complex than in minimal medium [41]. For further characterization, it would be important to determine NAME concentrations in cultures grown in defined media containing different carbon or nitrogen sources or with limited access to essential nutrients and cofactors like iron and sulfur.

### AHL- and NAME-spiked cultures exhibit different expression levels of stress response genes

Comparison of gene expression in NAME-, AHL- and AHL/NAME-spiked cultures revealed that under the tested conditions incubation time was an overall stronger impact factor than addition of either metabolite (Fig. 5). Major changes in the transcriptomes over time are therefore likely attributed to general changes in aging of the batch cultures including accumulation of harmful metabolic side and end-products or depletion of essential nutrients and substrates [55]. Only a small number of genes exhibited significantly differential expression between the conditions, when controlling for the effect of the growth time. Our results indicate that natural response mechanisms to these changing conditions over time are likely suppressed by regulation through C14:1-AHL. Two hours after the addition of external C14:1-AHL, several stress response genes in temporal expression cluster 2 were downregulated in AHL- or AHL/NAME-spiked cultures relative to the NAME-cultures. For instance, the oxidative stress response genes *hslVU* (PVU19_RS00735, PVU19_RS00740), *selO* (PVU19_RS10705) and superoxide dismutase (PVU19_RS08760) were downregulated in AHL- or AHL/NAME-cultures in the stationary phase (see Supplement F3). The stress response protease HslVU has previously been described to interfere with Fts cell division proteins and cell division inhibitor SulA to slow down cell division in the presence of large amounts of misfolded or unfolded proteins [56]. Thereby, damage repair is prioritized over cell division, e.g., under oxidative stress [57]. This is especially important at later growth stages when reactive oxygen species accumulate naturally as by-products of respiratory processes. We assume that the upregulation of stress response genes in NAME-cultures (relative to AHL- or AHL/NAME-cultures) reflects the natural transcriptional response towards the onset of the stationary phase and that C16:1-NAME has no impact on the expression of these stress response genes. This is supported by our observation that AHL/NAME-cultures exhibit the same transcriptional pattern as AHL-cultures, instead of an AHL/NAME -hybrid or intermediate pattern.

After addition of C16:1-NAME in the early exponential phase, the gene expression patterns between NAME-spiked and unspiked cultures were largely similar. We assume that there are several possible explanations for this absence of distinct NAME-specific patterns: Firstly, a generally high biological background variation (sample-to-sample variation) observed in our data may have masked any minor influences of C16:1-NAME on gene expression. Secondly, as C16:1-NAME is continuously produced by EL-164, genes transcriptionally affected by C16:1-NAME might already be expressed or suppressed by the constant presence of NAME in the culture and therefore not exhibit a visible response to the NAME spike in the transcriptomes. Finally, it has not been determined yet, if NAMEs are actively exported from the cell or, vice versa, can be taken up from the environment. Thus, the impact of external C16:1-NAME on EL-164 gene expression might be limited. In contrast to the signaling molecule AHL, external NAMEs might not enter EL-164 cells. While AHLs are transported in and out of the cell across the membrane [19] it is unknown whether NAMEs are actively secreted or could be actively taken up. Depending on the mechanism of NAME-uptake, intracellular concentrations might have been very low in our experimental setup, despite the high C16:1-NAME spike added to the EL-164 cultures. As stated above, comparisons between intracellular and extracellular NAME concentrations would provide further insights into such processes.

Several significantly differentially expressed genes in clusters 2 and 3 were only partially characterized as via COG categories, manual comparison with RefSeq database entries and GO terms. Multiple genes remained uncharacterized or were only basically classified as “biosynthetic”. Hence, we hypothesize that *R. tolerans* EL-164 may produce one or more so far undescribed metabolites. The different transcription patterns in NAME-spiked and AHL- or AHL/NAME-spiked cultures indicate that these genes are potentially regulated via AHL-mediated *quorum sensing*.

While we can conduct a relative comparison between NAME-, AHL/NAME- and AHL-spiked cultures, it must be noted that further experiments are needed to enable an absolute evaluation of either metabolite spike on EL-164 gene expression. Future experiments should include a solvent-treated and an untreated control group in addition to the metabolite treated cultures, to establish a transcriptomic “baseline”, as well as the systematic analysis of the metabolites at all compared time points.

## Conclusions

*R. tolerans* EL-164 exhibited distinct production patterns for NAMEs and AHLs, indicating different biological relevance of the two compounds. Both the observed production pattern and the calculated concentrations of C16:1-NAME in EL-164 cultures, which are higher than the MIC reported in previous studies, hint to antimicrobial activity as an ecological role. Moreover, C16:1- and C17:1-NAME were produced in markedly different quantities and possibly at different time points during growth. Considering the diversity of structural analogues of the NAMEs produced by EL-164, we assume that this strain has a high potential for so far undescribed bioactivities. The described NAME quantification method enables further dose-dependent screenings, thereby providing a basis for future studies to work with biologically relevant NAME concentrations.

## Electronic supplementary material

Below is the link to the electronic supplementary material.

Supplementary Material 1: Word document describing pre-experiments, NAME extraction procedures (Table T1) and cultivation of EL-164 for metabolite extraction and spike-in incubations.

Supplementary Material 2: Word document containing additional figures and tables with results from the quantification experiment (Figures F1-4 and Tables T1-2).

Supplementary Material 3: Excel file containing a table of all 187 significantly differentially expressed genes displayed in Figure 6 and the individual expression plots for each DE gene.

## Data Availability

The complete genome of *Roseovarius tolerans* EL-164 was deposited at GenBank (GCA_028885395.1). The dataset supporting the conclusions of this article is available in the Gene Expression Omnibus (GEO) repository, (entry GSE270994, comprising GSM8366822-GSM8366844, https://www.ncbi.nlm.nih.gov/geo/query/acc.cgi?acc=GSE270994).
