# Supplementary material for: Chemical quantification of N-acyl alanine methyl ester (NAME) production and impact on temporal gene expression patterns in Roseovarius tolerans EL-164
Source: BMC Microbiol. 2024 Nov 21;24:489. doi: 10.1186/s12866-024-03624-7 (PMC11580390; doi:10.1186/s12866-024-03624-7)
Supplement: Supplementary file 1 — Supplementary Material 1: Word document describing pre-experiments, NAME extraction procedures (Table T1) and cultivation of EL-164 for metabolite extraction and spike-in incubations. [file 12866_2024_3624_MOESM1_ESM.docx]

**Supplement F1 – Pre-experiments and experimental setups**

# Extraction of secondary metabolites from EL-164 cultures

In the following, the pre-experiments conducted to develop a suitable extraction method for NAMEs are described. It was tested whether an adsorbent should be used and when it should be added to the bacterial culture, as well as the optimum time point to add the internal standard (IS) **4** to the samples obtained from EL-164 cultures.

## Description of extraction procedures and conditions tested for addition of IS

### Solid phase extraction

The adsorbent Amberlite XAD-16 (Alfa Aesar, Kandel, Germany) was purified prior to its application. Therefore, the adsorbent was slurried three times in methanol, stirred vigorously for 30 min and decanted. Residual methanol was evaporated and the adsorbent was purified by Soxhlet extraction with acetonitrile and diethyl ether for four and two hours respectively, and finally dried under high vacuum. Two different NAME extraction procedures were compared: (A) addition of adsorbent before inoculation (incubation with the adsorbent throughout the total cultivation period) and (B) addition of adsorbent immediately before extraction (incubation for a designated time span in a sub-sample).

For (A), purified and autoclaved XAD-16 was added to bacterial cultures before inoculation (2 g adsorbent per 100 mL culture). Then, EL-164 was routinely cultivated. For sampling, complete cultures were harvested by filtering through a clean metal sieve retaining the adsorbent. The collected XAD-16 was extracted three times with a mixture of DCM and water (1:2). The DCM-water extraction mixture was collected in a separatory funnel. Before discarding the adsorbent, XAD-16 was washed again three times with pure DCM and the solvent was added to the separatory funnel. The organic phase was collected from the funnel and the aqueous phase was washed again with DCM to increase the extraction yield. The organic phases were pooled and dried with MgSO_4_ and filtered (Rotilabo, type 600P, Carl Roth, Karlsruhe, Germany). The filter cake was washed three times with excess DCM to increase the extraction yield. For the same purpose, throughout the extraction procedure, all glassware and devices were rinsed thoroughly with DCM, which was collected and pooled with the rest of the sample. The organic extract was concentrated in a rotary evaporator (Multivapor P-6, Büchi, Flawil, Switzerland) and stored at -20 °C until chemical analysis.

For (B), the bacterial culture was incubated under routine conditions without adsorbent. One hour before the desired extraction time point, XAD-16 (2 g resin per 100 mL culture) was added to the bacterial culture aseptically. Subsequent extraction was performed as described.

### Liquid-liquid extraction

For solvent extraction, the bacterial culture was grown under routine conditions. A sample was aseptically removed from the culture and immediately centrifuged (7,500 rpm, 4 °C, 30 min). Subsequently, the supernatant (SN) was sterile filtered (ø 2 µm, PES, Fisher Scientific, Waltham, MA, USA) and extracted with DCM by mixing in a separatory funnel (DCM:SN=1:3). The aqueous and the organic phase were collected separately. The aqueous phase was reextracted twice with DCM. After the third extraction step the aqueous phase was discarded and the collected organic extract was dried with anhydrous MgSO_4_ and filtered through a paper filter (Rotilabo, type 600P). The organic extract was concentrated in a rotary evaporator (Multivapor P-6, Büchi, Flawil, Switzerland) and stored at -20 °C until chemical analysis.

### Addition of the internal standard before and after incubation

The deuterated IS was added either (a) before the extraction procedure or (b) afterwards. For (a), the standard was added to the culture 1 hour prior to the designated extraction time point. Incubation was continued under standard conditions. Then, cultures were extracted as described via solid- or liquid-phase extraction. For (b), the IS was added just after the last extraction step and the sample was stored (dark, 4 °C) until NAME quantification via GC-MS.

## Comparison of results obtained using different NAME extraction procedures

NAME concentrations determined in EL-164 extracts prepared using XAD-16 were approximately four times higher than those obtained for extracts prepared without XAD-16 (**Table S1**). While high extraction quantities are often desirable, several aspects observed during the pre-experiments led to a preference of the adsorbent-free procedure over an XAD-based extraction in the main experiment: Firstly, sterile removal of a defined amount of XAD from a larger culture at several consecutive time points was not operable. Secondly, bacterial cells adhering to XAD-16 globules were lysed by the solvent, thus disabling discrimination between intra- and extracellular NAMEs. Thirdly, binding of extracellular NAME by XAD may have resulted in a positive feedback and potentially induced NAME overproduction in EL-164 to maintain extracellular metabolite concentrations.

Culture extracts supplemented with the IS immediately prior to extraction yielded approximately three times higher NAME concentrations than those receiving the IS after extraction (**Table S1**). Further, addition of the IS prior to the extraction procedure would reveal potential metabolite losses e.g., via adsorption to glassware which are detected in this way and can be corrected mathematically.

Table S1: NAME concentrations determined in *R.* *tolerans* EL-164 culture extracts prepared using different procedures. Values are means of NAME concentrations determined in extracts from three biological replicates. XAD - Amberlite XAD-16

| **Treatment** | **C16:1-NAME [µg L^-1^]** | **C17:1-NAME [µg L^-1^]** |
| --- | --- | --- |
| without XAD* | 158 | 37 |
| with XAD* | 701 | 87 |
|  |  |  |
| without XAD, standard after extraction | 633 | 153 |
| without XAD, before after extraction | 2005 | 223 |
| *internal standard for quantification added after extraction | | |

# Experimental setup 1: Cultivation of EL-164 for quantification of C16:1- and C17:1-NAME

Precultures of EL-164 were set up in 150 mL MB in baffled flasks and were incubated under standard conditions for 24 h. The main cultures were set up in 1,000 mL MB in baffled flasks with a starting OD_600nm_ of 0.03 and were incubated as before. Growth was monitored via OD_600nm_. The experiment was conducted in biological triplicates. Sampling time points were predefined as follows: immediately after inoculation (t_Q0_) and throughout the entire growth phase to cover the exponential (t_Q1,Q2_), deceleration (t_Q3_) and stationary (t_Q4,Q5_) phases of EL-164. For secondary metabolite extraction (by liquid-liquid extraction as described above), 150 mL samples were removed aseptically from each replicate and immediately processed. Simultaneously, 1 mL culture liquid was collected from each flask, fixed with 27 µL formaldehyde (38 %) and stored at -20 °C for later determination of bacterial cell numbers (described below). To enable downstream quantification of NAMEs in the organic extract, an IS was added (**4**, final concentration: 210 µg L^-1^) to each sample immediately after sampling and prior to centrifugation. To rule out contamination during repeated sampling for OD_600nm_ measurements and NAME extraction, cultures were checked for purity by frequent microscopic observations and repeated plating of randomized samples. Growth rates (µ, formula 1) were determined between OD_600nm_ measurements and used for differentiation between growth phases.

1. $\mu=\frac{ln(\frac{{OD}_{t(x+1)}}{{OD}_{tx}})}{t_{(x+1)}-t_{x}}$

## Cell counts

Samples taken for cell counts were thawed and vacuum-filtered on black mixed cellulose ester membrane filters (0.2 µm pore size; Merck Millipore, Burlington, MA, USA) and stained with SYBR green. Bacterial cells were counted using an Axioskop 2 microscope (Carl Zeiss, Jena, Germany).

## Statistical evaluation

Statistical analyses were performed using R (version 3.6.2) (R Core Team 2020) and RStudio (version 1.0.44). The data were tested for normality using the Shapiro–Wilk’s W-test and for homogeneous variances using Levene’s test. Welch's t-tests were used for pairwise comparisons between two successive time points and were performed to identify significant differences (p=0.05) between mean values obtained for cell numbers, AHL and NAME concentrations.

# Experimental setup 2: Cultivation of EL-164 for spike-in experiment, RNA extraction, sequencing and data analysis

The timing and concentration of the metabolite spikes was chosen based on the results obtained during the quantification experiment. All cultures were sampled for RNA extraction and sequencing in the mid exponential phase, after the addition of NAMEs to the designated cultures (t_S1_), as well as immediately prior to and after the AHL spike-in (t_S2_ and t_S3_ respectively).

## Cultivation, sampling and RNA extraction procedures

EL-164 was grown in 150 ml MB under standard conditions. The experiment was set up with an OD_600nm_ = 0.01 with three biological replicates (designated B-D) and four different test conditions: NAME-cultures were spiked with synthetic C16:1-NAME (0.8 mg/flask = 5.33 mg/L = 15.72 µM) during early exponential phase, when C16:1-NAME-concentrations in EL-164 cultures were, based on results from the quantification experiment, expected to be low. AHL-cultures were spiked with synthetic C14:1-AHL (11.03 mg/flask = 73.53 mg/L = 0.238 mM) in the deceleration phase, when AHL concentrations in cultures of the quantification experiment were dropping. AHL/NAME-cultures were spiked with both metabolites as described above (125 µl/flask = 0.083 vol% and 250 µl/flask = 0.167 vol% for NAMEs and AHLs respectively).

For sampling, 2 ml bacterial culture was obtained aseptically, snap frozen in liquid nitrogen and subsequently stored at -80 °C until RNA extraction. The latter was performed as described previously (1), omitting the DNA extraction steps.

# References

1. Leinberger J, Milke F, Christodoulou M, Poehlein A, Caraveo-Patiño J, Teske A, et al. Microbial epibiotic community of the deep-sea galatheid squat lobster Munidopsis alvisca. Scientific Reports 2022 12:1. 2022 Feb 17;12(1):1–15.
