# Supplementary material for: Chemical quantification of N-acyl alanine methyl ester (NAME) production and impact on temporal gene expression patterns in Roseovarius tolerans EL-164
Source: BMC Microbiol. 2024 Nov 21;24:489. doi: 10.1186/s12866-024-03624-7 (PMC11580390; doi:10.1186/s12866-024-03624-7)
Supplement: Supplementary file 2 — Supplementary Material 2: Word document containing additional figures and tables with results from the quantification experiment (Figures F1-4 and Tables T1-2). [file 12866_2024_3624_MOESM2_ESM.docx]

**Supplement F2 – Additional figures and tables**

# Detection and quantification of NAMEs and AHLs produced by *R. tolerans* EL-164


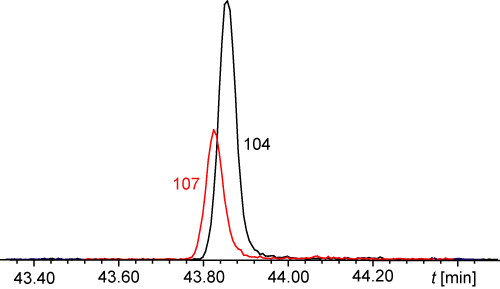


Figure S1: Overlay of single ion chromatograms of the internal standard 4 (m/z 107) and the natural C16:1-NAME (5, m/z 104). The observed shift in elution time is due to the shorter C-D-bond length in 4 compared to the C-H bond length in the natural compound.


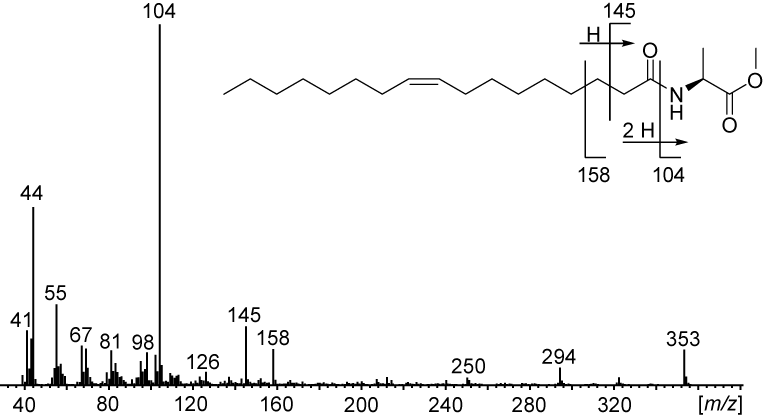


Figure S2: Mass spectrum and fragmentations of the natural metabolite C17:1-NAME (6).


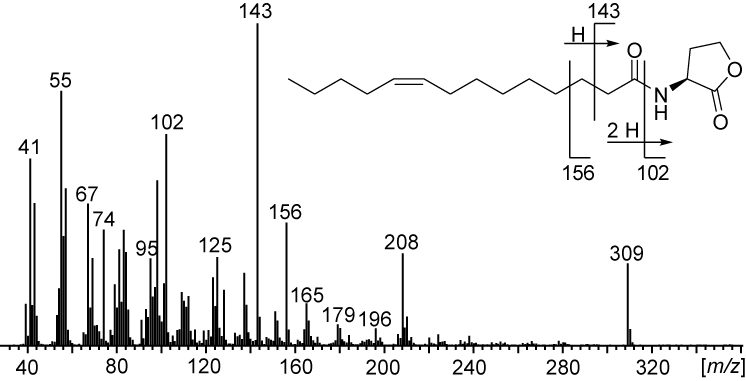


Figure S3: Mass spectrum and fragmentations of the natural metabolite C14:1-AHL (7).

## Growth characteristics of *Roseovarius tolerans* EL-164

Table S1: Growth parameters of *R. tolerans* EL-164 determined under routine culture conditions. NAMEs and AHLs were extracted via liquid-liquid extraction and measured with GC/MS. The internal standard (C16:1-NAME-d3, 4) was added to each subsample directly before the extraction procedure. Growth rates were calculated according to formula (1) provided in Supplement F 1. All given values are means calculated from biological triplicates. n.d. - not determined

| **Time point** | **t_Q0_** | **t_Q1_** | **t_Q2_** |  |
| --- | --- | --- | --- | --- |
| Incubation time [h] | 0 | 21 | 30 |  |
| OD_600nm_ | 0.03 ± 0.00 | 0.18 ± 0.02 | 0.40 ± 0.04 |  |
| Cell counts [cells L^-1^] | n.d. | 7.45 x 10^-11^ ± 1.17 x 10^-11^ | 1.44 x 10^-12^ ± 4.33 x 10^-10^ |  |
| Growth rate [h^-1^] | n.d. | 0.091 ± 0.004 | 0.091 ± 0.003 |  |
| Growth phase | inoculation | exponential | exponential |  |
| C16:1-NAME [µg L^-1^] | no | 684.9 ± 79.4 (2.0 ± 0.2 µM) | 1,101.1 ± 304.1 (3.2 ± 0.9 µM) |  |
| C16:1-NAME-d_3_ [µg L^-1^] | missing | 210 | 210 |  |
| C17:1-NAME [µg L^-1^] | no | 0.0 ± 0.0 | 5.3 ± 9.2* (15 ± 26 nM) |  |
| C14:1-AHL [mg L^-1^] | yes | 58.7 ± 12.4 (189.7 ± 40.1 µM) | 35.9 ± 12.5 (116 ± 40.4 µM) |  |
| *compound found only in one replicate (of three) | | | | |

Table S1: Continued.

| **Time point** | **t_Q3_** | **t_Q4_** | **t_Q5_** |
| --- | --- | --- | --- |
| Incubation time [h] | 48 | 81 | 105 |
| OD_600nm_ | 0.86 ± 0.04 | 1.90 ± 0.09 | 2.14 ± 0.16 |
| Cell counts [cells L^-1^] | 3.28 x 10^-12^ ± 2.06 x 10^-11^ | 6.47 x 10^-12^ ± 5.11 x 10^-11^ | 8.03 x 10^-12^ ± 2.03 x 10^-12^ |
| Growth rate [h^-1^] | 0.070 ± 0.001 | 0.050 ± 0.001 | 0.039 ± 0.001 |
| Growth phase | deceleration | early stationary | stationary |
| C16:1-NAME [µg L^-1^] | 2,931.3 ± 386.8 (8.6 ± 1.1 µM) | 4,364.0 ± 640.2 (12.9 ± 1.9 µM) | 5,731.8 ± 851.6 (16.9 ± 2.5 µM) |
| C16:1-NAME-d_3_ [µg L^-1^] | 210 | 210 | 210 |
| C17:1-NAME [µg L^-1^] | 28.2 ± 3.2 (79.8 ± 9.1 nM) | 47.3 ± 7.5 (133.8 ± 21.2 nM) | 86.4 ± 8.7 (244.3 ± 24.6 nM) |
| C14:1-AHL [mg L^-1^] | 17.5 ± 4.2 (56.6 ± 13.6 µM) | 19.7 ± 3.6 (63.7 ± 11.6 µM) | 21.4 ± 6.3 (69.2 ± 20.4 µM) |
| *compound found only in one replicate (of three) | | | |


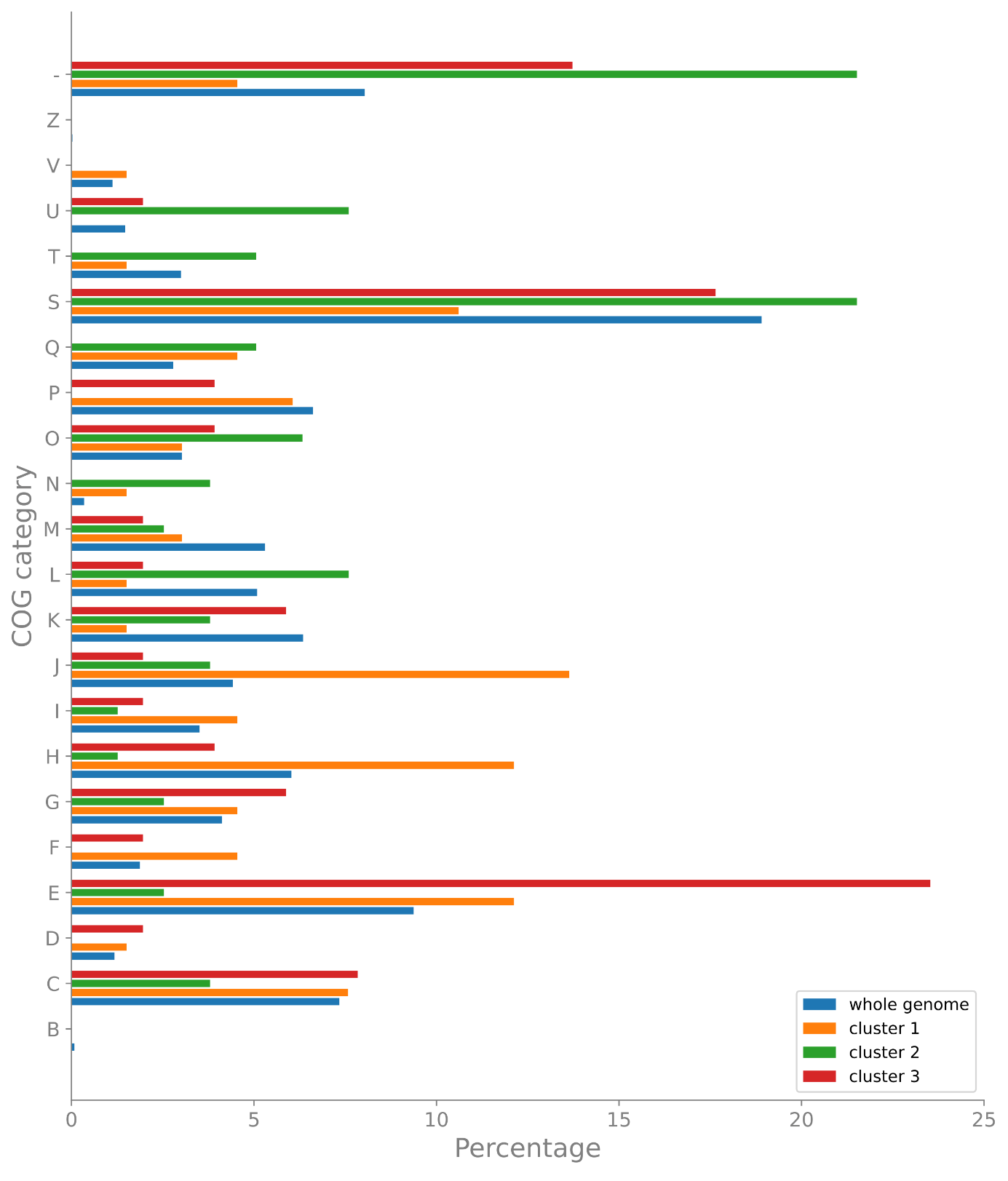


Figure S4: Distribution of COG categories in the whole genome of *R. tolerans* EL-164 and in the clusters 1-3 comprising differentially expressed genes. Categories were assigned using eggNOG mapper. Abbreviations are as follows: - no category assigned, Z “Cytoskeleton”, V “Defense mechanisms”, U “Intracellular trafficking”, “secretion and vesicular transport”, T “Signal transduction mechanisms”, S “Function unknown”, Q “Secondary metabolites biosynthesis, transport and catabolism”, P “Inorganic ion transport and metabolism”, O “Posttranslational modification, protein turnover, chaperones”, N “Cell motility”, M “Cell wall/membrane/envelope biogenesis”, L “Replication, recombination and repair”, K “Transcription”, J “Translation, ribosomal structure and biogenesis”, I “Lipid transport and metabolism”, H “Coenzyme transport and metabolism”, G “Carbohydrate transport and metabolism”, F “Nucleotide transport and metabolism”, E “Amino acid transport and metabolism”, D “Cell cycle control, cell division, chromosome partitioning”, C “Energy production and conversion”, B “Chromatin structure and dynamics
